# Perceptions of Care Among Older Adults Identified as Fall Risk During a Hospital Stay

**DOI:** 10.1093/geroni/igaa057.455

**Published:** 2020-12-16

**Authors:** Barbara King

**Affiliations:** University of Wisconsin-Madison, Madison, Wisconsin, United States

## Abstract

In-hospital falls are a significant clinical, legal and regulatory problem. The Centers for Medicare and Medicaid no longer reimburse hospitals for falls that result in injury, adding increase pressure on acute care settings to prevent falls. Additionally, evidence-based practice recommendations for fall prevention in hospitals do not exist, thus leaving administrators to create their own programs. One common strategy used by hospital providers to prevent falls is to restrict patient mobility. Little information on how older adult patients experience fall prevention during a hospital stay has been published. The purpose of this study was to understand perceptions of care among older adults identified as fall risk during a hospital stay. This qualitative study utilized inductive content analysis. Older adults (N=20) from a large academic medical center in the Midwest were recruited to participate in one-to one in-depth interviews. Open coding, categorization and abstraction was used to analyze the data. Three main categories were identified that summarized the older adult patient perception of hospitalization: Act of Caring, something they received from staff, provide to staff or provided to self; Being Restricted in movement resulting in either accepting or rejecting the restriction and Being Freed at discharge, often being told “just be careful”. Older adult identified as fall risk described being restricted in movement during a hospital stay. Many passively accepted this restriction even though they felt a lack of movement would be harmful to them. Additional research on the patient experience with fall prevention is needed.

